# Yes-Associated Protein and Transcriptional Coactivator with PDZ-Binding Motif in Cardiovascular Diseases

**DOI:** 10.3390/ijms24021666

**Published:** 2023-01-14

**Authors:** Ruojun Li, Weiqiang Huang

**Affiliations:** Department of Geriatric Cardiology, Guangxi Key Laboratory Base of Precision Medicine in Cardio-Cere Brovascular Diseases Control and Prevention, Guangxi Clinical Research Center for Cardio-Cerebrovascular Diseases, The First Affiliated Hospital of Guangxi Medical University, Nanning 530021, China

**Keywords:** YAP, TAZ, cardiovascular diseases, myocardial infarction, atherosclerosis, CHD

## Abstract

Yes-associated protein (YAP, also known as YAP1) and its paralogue TAZ (with a PDZ-binding motif) are transcriptional coactivators that switch between the cytoplasm and nucleus and regulate the organ size and tissue homeostasis. This review focuses on the research progress on YAP/TAZ signaling proteins in myocardial infarction, cardiac remodeling, hypertension and coronary heart disease, cardiomyopathy, and aortic disease. Based on preclinical studies on YAP/TAZ signaling proteins in cellular/animal models and clinical patients, the potential roles of YAP/TAZ proteins in some cardiovascular diseases (CVDs) are summarized.

## 1. Introduction

According to the American Heart Association, although the mortality rate of cardiovascular diseases (CVDs) has decreased in recent years, it is still one of the major causes of death worldwide. In addition, the ageing of the population has led to increased CVDs and an increased cardiovascular burden in developing countries, resulting in socioeconomic damage [1,2]. Therefore, enriching the therapeutic approaches and strategies for CVDs has important prospects.

Yes-associated protein (YAP, also known as YAP1) is a classic downstream effector in the Hippo signaling pathway and is an important factor that regulates cell proliferation and differentiation [3]. The transcriptional coactivator YAP, which acts as a shuttle between the cytoplasm and nucleus, and its smaller paralogue TAZ (a transcriptional coactivator with a PDZ-binding motif) are key regulators of the organ size and tissue homeostasis. YAP localizes to the nucleus in its dephosphorylated form and binds to transcription enhancer-associated domain 1–4 transcription factors (TEAD1–4) to drive the downstream gene expression [4,5,6]. The dysregulation of these factors can lead to a variety of human diseases [7]. The phosphorylation of YAP/TAZ promotes a cytoplasmic localization, thereby inhibiting the YAP/TAZ transcriptional activity. When the Hippo pathway is activated, the YAP/TAZ protein is phosphorylated by the large tumor suppressor kinase 1/2 (LATS1/2) at five serine residues, leading to nuclear exclusion, ubiquitination, and a subsequent protein degradation [8]. In addition, YAP/TAZ proteins, which are transcriptional coactivators, also interact with other transcription factors, such as the SMAD family member 7 (SMAD7), receptor protein-tyrosine kinase ErbB-4 (ErbB4), and activator protein-1 (AP-1), to control the expression of the corresponding target genes [9,10,11,12,13]. Independent of the Hippo pathway, YAP/TAZ can be activated by actin polymerization and Rho GTPase [14,15] and can also interact with actin-sensitive myocardin-related transcription factor (MRTF) [16,17].

**Figure 1:**
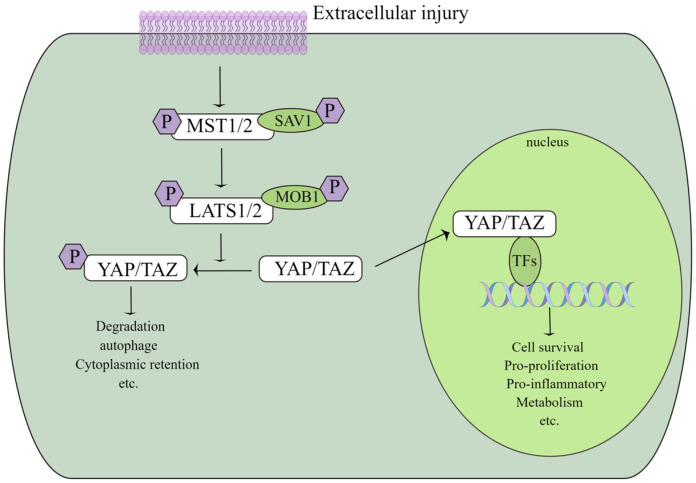
Mechanism of the YAP/TAZ signaling protein.

Many recent studies have reported that YAP/TAZ plays a significant role in the development of CVDs, including pulmonary hypertension, atherosclerosis, aortic disease, and angiogenesis [18,19,20,21,22]. Here, we discuss the relevant role of the YAP protein in certain CVDs. Figure 1 shows the mechanism of the YAP/TAZ signalling proteins.

## 2. YAP/TAZ in Myocardial Infarction (MI) and Cardiac Repair after MI

An acute MI is myocardial necrosis caused by the acute and persistent ischaemia and hypoxia of the coronary artery, which can be complicated by arrhythmia and heart failure and is often life-threatening [23]. Different cells of the heart after an MI can improve the ventricular remodeling, reduce the MI size, reduce the myocardial cell apoptosis, and inhibit myocardial fibrosis through various ways, thereby improving the cardiac function after an MI [24].

### 2.1. YAP/TAZ in Cardiomyocytes (CMs)

Cardiomyocytes are the main effectors of a myocardial contraction, and changes in the CMs proliferation or myocardial hypertrophy are an important part of the recovery of the cardiac function after an MI [25]. The proliferation of CMs during development is regulated by a variety of growth factors, such as insulin-like growth factors (IGFs) and neuregulin [26]. However, the cell-intrinsic pathway mechanisms that regulate the CMs proliferation remain unclear. LncRNAs and miRNAs have recently been recognized to play key roles in the cardiac development, physiological function, and pathological processes [27,28,29,30]. miRNAs can act as posttranscriptional regulators to degrade or translationally repress mRNAs and downstream proteins, thereby inhibiting the expression of target genes and regulating the cell growth, proliferation, and survival in cardiac pathophysiology [31]. The YAP can regulate various transcription factors, including the TEAD, Runt-related transcription factor (RUNX), and T-box transcription factor 5 (TBX5) [32,33,34]. By measuring the level of cytoplasmic and nuclear YAP in CMs treated with different miRNAs, some researchers proved that miR-199a-3p, the miR-302 family, and other pro-proliferation miRNAs directly or indirectly down regulate the cell kinases related to regulating the YAP through different ways, such as TAO kinase 1 (TAOK1) and serine/threonine kinase 38 like (STK38L), which is mainly achieved by regulating the levels of different effector genes [35]. miRNAs such as miR-590-3p, miR-1825, miR-1248, miR-18a, miR-33b, and miR—30e were also included in the research queue; also, miR302-367 clusters can promote cardiomyocyte proliferation by regulating the Hippo pathway to inhibit the expression of LATS2 [36]. A recent study showed that LncDACH1 directly binds to protein phosphatase 1 catalytic subunit alpha (PP1α), thereby limiting its dephosphorylation activity. The upregulation of LncDACH1 attenuates a cardiac regeneration, promotes the phosphorylation of the YAP, and reduces its nuclear translocation, thereby inhibiting the CMs proliferation and reactivating cardiac regeneration [37]. The increase in the YAP nuclear activity is a necessary condition to promote these miRNAs to induce CMs proliferation. In addition, their other common way of action is to participate in the regulation of the formation of cytoskeleton by balancing Filamentous actin (F-actin) and Globular actin (G-actin), which is precisely the strong activation signal of the YAP in response to the mechanical signals generated by the extracellular environment [38]. However, the pleiotropic function of miRNA has not been stably exerted. For example, the permanent expression of miR-302/367 clusters can lead to an excessive proliferation and the cell dedifferentiation of CMs in transgenic animals, leading to cardiac dysfunction [36].

In addition, miRNA is also an important downstream effector of YAP. In addition, the forkhead box transcription factor (FoxP1) is a physiological mediator of the miR-206 function in CMs and is also an endogenous negative regulator of cardiac hypertrophy and survival. As an important downstream effector, the YAP upregulates the expression of miR-206 and regulates the CMs function by silencing the FoxP1 in CMs [39]. Notably, the typical binding sites for the YAPs transcriptional partners, such as TEADs, RUNX, ErbB4, Tbx5, and Smad, are not contained in the enhancer region of miR-206 [11,40,41,42]. However, the identity of this E-box-binding transcription factor and its functional relationship to the YAP remain to be confirmed. In addition, studies have also shown that miRNAs mediate the other nongrowth-related functions of the YAP. For example, the YAP upregulates the miR-29s expression, which in turn inhibits the phosphatase tensin homologue (PTEN) and activates the mammalian target of the rapamycin (mTOR) pathway [43]. Cardiac fibroblasts have enriched miR-29, which suppresses cardiac fibrosis [44]. However, the overexpression of miR-29 does not induce CMs hypertrophy in neonatal rats [39]. Alternatively, miR-484 directly targets the YAP to suppress the cell viability and promote the NOD-like receptor thermal protein domain-associated protein 3(NLRP3) inflammasome and the production of the inflammatory cytokines tumor necrosis factor-α (TNF-α), interleukin-1β (IL-1β), and interleukin-6 (IL-6), increasing apoptosis in H9c2 cells [45].

YAP/TAZ can target some lncRNAs and miRNAs to mediate upstream and downstream targets or pathways to regulate the biological activity and cellular function; the increase in its nuclear translocation is conducive to the CMs proliferation [30,46]. However, do the YAP/TAZ proteins act only through a specific pathway or a combination of pathways? Do these pathways in turn regulate single or multiple mRNA targets? To what extent do they affect the CMs proliferation? These questions remain to be answered.

### 2.2. YAP/TAZ in Endothelial Cells (ECs)

In an endothelial hypoxia model, silencing the YAP impaired the hypoxia-induced ECs proliferation and angiogenesis, while the endothelial heat shock protein 12B (HSPA12B) bound to the YAP and acted as a target and coactivator of the YAP/TEAD4 to regulate endothelial angiogenesis after an MI, hypoxia makes HSA12B translocate to the nucleus, which is accompanied by the YAPs activation and increased nuclear translocation of endothelial cells. The interaction between the two synergistically regulates angiogenesis. The possible mechanism is that HSPA12B forms clusters with YAP/TEAD4, then the YAP is positively activated at the transcriptional level after hypoxia and the YAP degradation is eliminated [47]. In fact, ECs are constantly affected by shear stress under the impact of blood flow. As a mechanical sensor sensitive to shear stress, the YAP/TAZ demonstrates activity changes along with the change in the blood flow mode, which is crucial for maintaining cell stability [48]. It has been proved that unidirectional shear stress increases the YAPs phosphorylation and inhibits the YAPs activity by activating integrin [20]. Unidirectional shear stress exists in normal straight blood vessels. Does that mean that the oscillatory shear stress generated in damaged blood vessels inhibits the phosphorylation of the YAP, increases the activity of the YAP, and promotes an inflammatory reaction and aggravates atherosclerosis? A study shows that in the hypoxia/reoxygenation (H/R) cell model, shear stress promotes the YAPs nuclear translocation through the YAP/miR-206/programmed cell death factor 4 (PDCD4) signal pathway, thereby inhibiting the apoptosis of myocardial microvascular endothelial cells, and also regulates a microvascular relaxation by increasing the expression of the platelet endothelial cell adhesion molecule-1 (PECAM-1) protein and phosphorylated endothelial nitric oxide synthase(p-eNOS) [49]. In addition, unidirectional shear stress also reduces the JNK signal transduction and inflammatory gene expression, reduces the monocyte adhesion on endothelial cells, and inhibits the formation of atherosclerosis [20]. In addition, an increase in the miR-93s expression contributes to cardiac angiogenesis in ischemic hearts by regulating the YAP/TAZ and LATS2 expression, protecting ECs from an H/R injury. The knockdown of LATS2 can reduce the ECs apoptosis and increase the ECs migration and endothelial vascularization, while the overexpression of the YAP can enhance the angiogenic activity of ECs [50]. Moreover, the overexpression of miR-93 significantly prevented the expression of the MI-induced intercellular adhesion molecule-1 (ICAM-1) and vascular cell adhesion molecule-1 (VCAM-1). The adhesion molecules ICAM-1 and VCAM-1 are highly expressed in activated ECs and play a powerful role in promoting the inflammatory cell crossing of the endothelium and myocardial infiltration [51,52]. The knocking down of the YAP/TAZ can also significantly reduce the expression of VCAM1 induced by TNF-α [53], which suggests that the endothelial YAP/TAZ may assist a vascular repair by regulating the inflammatory reaction. The dephosphorylation/activation of the endothelial YAP/TAZ signal in a high glucose microenvironment is considered as the key link [54]; this will increase the endothelial inflammation and monocyte attachment [53,55].

Angiogenesis after an MI is an important step in cardiac repair. As a shear stress-sensitive mechanical sensor, the YAP/TAZ activity changes with different blood flow patterns. The YAPs activation and nuclear translocation of ECs play a protective role in the function of ECs in the cardiac dysfunction after a myocardial infarction. Paradoxically, changes in the activity and location of the endothelial YAP/TAZ will affect the inflammatory microenvironment. Most studies believe that the increased nuclear translocation of endothelial YAP/TAZ will aggravate the progress of the inflammatory response.

### 2.3. YAP/TAZ and Hormones

The YAP/TAZ may also be involved in cardiac repair after an MI through an endocrine mediation [52]. Adult cardiomyocytes have no regenerative capacity, and the hormones involved in development, metabolism, and maintaining homeostasis may play an indispensable role in it. Studies have shown that the loss of a cardiac regeneration in adult mammals is accompanied by an increase in thyroid hormones [56]. For example, the increased expression of the YAP in progesterone-treated CMs is associated with the activation of the YAP target genes (including the ankyrin repeat domain protein 1 (Ankrd1), connective tissue growth factor (CTGF), and cysteine-rich angiogenesis-inducing factor 61 (CYR61)), suggesting that a progesterone supplementation enhances the CMs proliferation in a progesterone receptor-dependent manner [57]. Melatonin promotes the CMs proliferation by inhibiting the miR-143-3p-mediated upregulation of the YAP and Catenin delta 1(Ctnnd1) by activating the melatonin receptor, but this has no effect on the cardiac fibroblast replication. In this study, both the YAP and Ctnnd1 were identified as the direct targets of miR-143-3p, but the relationship between Ctnnd1 and YAP was not clearly elucidated. Ctnnd1 may activate the Wnt/β-catenin signaling pathway and promote the formation of β-catenin and YAP complexes [58]. These studies suggest that changes in the circulatory environment, especially the hormone levels, may have an important impact on the cardiac regulation after an MI. However, the continuous activation of the YAP may lead to unfavorable adaptive cardiac hypertrophy and lead to the occurrence of body tumors [59,60]. How to adjust the time and dose of hormone targeting and reduce the side effects is a future direction for exploration.

### 2.4. YAP/TAZ and Fibroblasts

Indeed, cardiac fibroblasts, which are the most prevalent cell type in the heart and the main source of the extracellular matrix (ECM), play an important role in regulating cardiac fibrosis after an MI. Studies have demonstrated that Erbb2 drives mechanotransduction signals during cardiac regeneration in mice and downregulates the YAPs activation signals based on cytoskeletal remodeling and phosphorylation, thereby promoting changes in the epithelial-mesenchymal transition (EMT), including the production of substantial amounts of ECM, cytoskeletal remodeling, and CMs migration [61]. In addition, studies have also confirmed the role of the ERK-YAP signal transduction mechanism. The binding of exogenous Agrin to the dystrophin-glycoprotein complex (DGC) promotes a conformational change, leading to the partial disassembly of the aggregated protein. The DGC acts as a mechanical bridge between the myocardial contractility mechanisms and the cardiac microenvironment, leading to a myofibrillar breakdown and activation of the YAP and ERK, thereby promoting the differentiation of mouse- and human-induced pluripotent stem cells into CMs [62]. Excessive cardiac fibrosis leads to stiff ventricles and severe heart failure [63,64]. The transition of myocardial fibroblasts to myofibroblasts is closely related to adverse ECM remodeling and scarring after an MI. Spatial changes in the collagen organization modulate the cardiac fibroblast phenotypic changes through the mechanical activation of p38-YAP-TEAD signaling [65]. The YAP can be highly expressed and have an increased nuclear localization in cardiac fibroblasts in response to a rigid matrix; furthermore, the YAP pathway may be involved in ECM remodeling as an important signal downstream of the angiotensin II type 1 receptor (AT1R) via mechanotransduction, but the specific mechanism remains unclear [66]. The deletion of the YAP/TAZ in cardiac fibroblasts ameliorates adverse cardiac remodeling after an MI by regulating the fibroblast proliferation and transdifferentiation into myofibroblasts and by regulating the cardiac fibroblast activation and fibroinflammatory responses downstream of the Wnt and TGF-β signaling pathways [67]. These results suggest that the interaction between the ECM and YAP plays an important role in cardiac repair after an MI.

### 2.5. YAP/TAZ and Immune Cells

Usually, ischemic or nonischemic CVDs pathologically manifests as the stress-mediated activation of innate immune signaling pathways in CMs [68], and stimulates the release of proinflammatory cytokines and the production of reactive oxygen species (ROS) [69]. Macrophages are known to be the primary immune cells that regulate proinflammatory and anti-inflammatory/reparative responses during the inflammatory and repair phases of cardiac repair, and an early inflammatory activation is an important event in the transition from an MI injury to later cardiac remodeling [70]. M1 macrophages often play a dominant proinflammatory role in the early stage of cardiac repair after an MI, and it is worth noting that the YAP/TAZ activation-mediated immune repair may play a negative role in cardiac repair after an MI. Studies have shown that the YAP/TAZ can act as both an activator in proinflammatory macrophages and a repressor in repair macrophages. The YAP/TAZ can promote the proinflammatory response by increasing the expression of IL-6 and reducing the expression of arginase 1(Arg1) by interacting with the histone deacetylase 3 (HDAC3)—nuclear receptor corepressor 1 (NCoR1), thereby enhancing MI-induced cardiac fibrosis and remodeling. It is worth noting that this study also shows that a YAP/TAZ deficiency promotes the reparative macrophage phenotype [71]. In contrast, M2 macrophages predominate and promote a cardiac fibrotic repair after the first week of a myocardial injury, possibly as a protective mechanism against a cardiac rupture [72,73]. A study showed that the YAP/TAZ suppressed the postinfarction inflammatory response in the epicardium by recruiting regulatory T cells (Tregs). Mice lacking an epicardial YAP and TAZ develop severe pericardial inflammation and myocardial fibrosis after an MI, whereas the local targeted injection of interferon-γ (IFN-γ) after an MI reduced the Treg infiltration into the injured myocardium in YAP/TAZ mutants and reduced myocardial fibrosis [74]. Studies have also shown that the YAP/TAZ is a key suppressor of Toll-like receptor 4 (TLR4)-mediated innate immune responses in CMs. TLR4/NF-κB signaling is activated in YAP-knockout hearts, and the cardiac shock induced by lipopolysaccharide is exacerbated, but the underlying mechanism by which YAP inhibits TLR4/NF-κB signaling has not been revealed [75]. In non-cardiac conditions, inhibiting intracellular innate immune signaling may be independent of the YAPs transcriptional activity [76]. In contrast, the YAP directly blocks the activity of the key innate immune signaling components, such as the interferon regulatory factor 3(IRF3), transforming growth factor β-activated kinase 1(TAK1), and Tank binding kinase 1(TBK1) [77,78,79]. These findings suggest that YAP/TAZs can be therapeutic targets for modulating immune cell phenotypes in disease states. Most of the existing studies focus on the role of the YAP/TAZ in macrophages, but few focus on the other types of immune cells. Future studies need to examine the role of the YAP/TAZ in immune signaling and clarify whether the YAP/TAZ-mediated immune regulation plays a protective/injurious role in disease recovery and whether the YAP/TAZ modulates the innate immune response in CMs through changes in its transcriptional activity or both.

In conclusion, the increase in the YAP/TAZ protein levels and its nuclear translocation can have a positive effect on cardiac repair after an MI. Activating the YAP can promote a myocardial cell proliferation, reconstruct blood circulation, improve the cardiac function, and reduce cardiac fibrosis. However, in the same disease model, protective/injurious effects can be different due to different expression levels or the targeting of different cardiac cells. This is a direction worth exploring in future research. Table 1 shows targeting different cells or cellular constituents of YAP/TAZ signaling proteins in myocardial infarction.

## 3. YAP/TAZ in Atherosclerosis and Coronary Heart Diseases

Hypertension, atherosclerosis, and coronary heart disease are common in CVDs, and their pathological features include the endothelial dysfunction caused by the impaired vascular function and structural abnormalities [82,83]. How the YAP/TAZ proteins are involved in the pathophysiology of this disease pathway remains unclear.

The long-term poor control of blood pressure in hypertensive patients can increase the pressure on the arterial vessel wall, damage ECs, and easily enter the arterial wall, stimulating the proliferation of vascular smooth muscle cells (VSMCs) and causing atherosclerosis, thereby causing coronary heart disease [84,85]. However, the mechanistic link between pathological changes and the elevated blood pressure-associated pathological changes in the vessel wall remains unclear. A normal contractile function and adequate mechanotransduction in the vascular smooth muscle are believed by some to play a key role in maintaining vascular integrity during elevated systemic blood pressure, and the YAP/TAZ are the mediators and sensors of the mechanical signals in the cellular microenvironment [38]. Endothelial YAP has been shown to be an important regulator of shear stress-induced atherosclerosis’ development and angiogenesis in mice [22,86]. Disheveled 3 (DVL3) increases nuclear translocation and upregulates osteogenesis-related genes in VSMCs in the absence of the YAP/TAZ, turning these cells towards osteogenic differentiation and vascular calcification after losing the differentiated phenotype [87]. Similarly, in a knockout mouse model, YAP/TAZ-deficient arteries were shown to have a reduced contractility, reduced stress-induced myogenic responses, and abnormal expression of the related genes. In addition, the YAP/TAZ knockout caused nonnegligible vascular damage in mesenteric arterioles, which indicates the protective effect of YAP/TAZ on hypertensive vascular disease [88].

In contrast, YAP/TAZ is closely related to the occurrence of the atherosclerotic inflammatory response, and inhibiting inflammation can delay the development of atherosclerosis [89]. The role of the YAP/TAZ signaling proteins in the pathological process of atherosclerosis is unclear. The Piezo-type mechanosensitive Ion channel component 1 (Piezo1) protein has been shown to play an important role in the inflammatory response in hypertension and some diseases [90,91]. Studies have shown that the activation of YAP/TAZ is accompanied by the oxidized low-density lipoprotein (ox-LDL)-induced enhancement of the Piezo1 activity in ECs, thereby mediating endothelial atherogenic inflammatory responses [92]. Second, ox-LDL can inhibit the normal expression of the YAP through the expression of miR-496, reduce the nuclear translocation of the YAP, and induce vascular ECs dysfunction [93]. Notably, the Ras homologous gene family member A (RhoA) is a critical regulator of Hippo signaling that responds to exogenous stimuli, such as cell fusion and mechanotransduction [14,94]. It has been suggested that the YAP/TAZ is indispensable for signaling in the response to shear stress and promotes atherosclerosis in response to the disturbed flow, which is dependent on RhoA-mediated signaling [89]. Studies have shown that the junctional cadherin 5-related protein (JCAD) interacts with LATS2 via RhoA, resulting in an increased YAP activity and ECs dysfunction, leading to atherosclerosis [95]. In addition, the YAP in macrophages acts as an IL-1β-triggered inducer through the tumor necrosis factor-associated factor-6 (TRAF6)-mediated ubiquitination of Lys63 and the subsequent destruction of angiomotin (AMOT) binding, thereby promoting the release of chemotactic mediators and accelerating the development of atherosclerosis [96,97].

**Figure 2:**
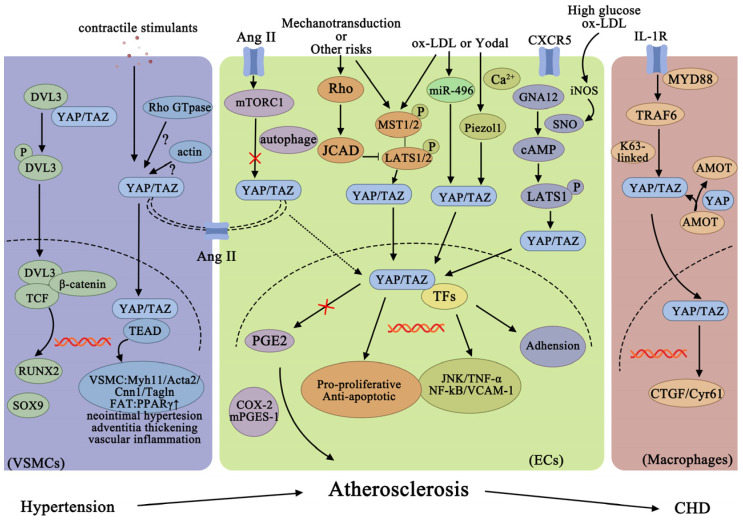
The role of YAP/TAZ of VSMCs, ECs, and macrophages in atherosclerosis and coronary heart disease. TCF, ternary complex factor; SOX9, SRY-box transcription factor 9; MYH11, muscle myosin heavy-chain 11; Acta2, actin alpha 2; Cnn1, calponin 1; Tagln, 22-kDa SMC lineage-restricted protein; PPARγ, peroxisome proliferator-activated receptor gamma; ANG II, angiotensin II; PGE2, prostaglandin E2; COX2, cyclooxygenase 2; m-PGES-1, microsomal prostaglandin E synthase-1; TFs, transcription factors; i-NOS, inducible nitric oxide synthase; GNA12, guanine nucleotide-binding protein subunit alpha-12; SNO, S-nitrosylation; CXCR5, C-X-C motif chemokine receptor 5; MYD88, myeloid differentiation factor 88; K63-linked, lysine-63 (K63)-linked.

In the heart and vascular system, mechanical mechanics affects the shape and physiological function of the heart. Laminar flow plays an important role in maintaining endothelial cell homeostasis, while turbulent blood flow leads to atherosclerosis. Whether YAP/TAZ regulates changes in the cell morphology, cytoskeleton, and ECM by mediating hemodynamics or inflammatory factors needs further study. All these indicate that targeting the YAP/TAZ signaling protein may be a new strategy to improve atherosclerosis. Figure 2 shows the role of YAP/TAZ of VSMCs, ECs, and macrophages in atherosclerosis and coronary heart disease.

## 4. YAP/TAZ in Cardiomyopathy

Cardiomyopathy is a kind of heart disease with a complex etiology, and basic lesions occur in the myocardium, resulting in an abnormal contraction in the myocardium [95]. Cardiomyopathy usually refers to primary cardiomyopathy, including hypertrophic cardiomyopathy (HCM), dilated cardiomyopathy (DCM), arrhythmogenic cardiomyopathy (AC), and restrictive cardiomyopathy (RCM) [98].

HCM is an autosomal dominant disorder that causes a sudden cardiac death in young adults [97]. This condition is characterized by unexplained left ventricular hypertrophy and microscopic myocyte disorganization and fibrosis [98]. A study showed that YAP was highly expressed and activated in HCM patient tissue samples. In a mouse HCM model, PI3K/Akt signaling was activated and inhibited the transcriptional activity of the forkhead box protein O3 (FOXO3), reducing FOXO3 binding to the mammalian sterile 20-like kinases 1 (MST1) promoter, and downregulating the MST1 expression levels, which led to a YAP transactivation and increased the expression of the thick filament component β-cardiac myosin heavy chain 7 (Myh7) and the thin filament component cardiac troponin 2 (Tnnt2), which are hallmarks of HCM. Furthermore, activated YAP feedback enhances the AKT activation, thereby forming a feedforward loop between the PI3K-AKT and MST1/YAP pathways [99]. However, the mechanism by which the YAP is transcriptionally downstream during HCM remains unknown.

DCM is characterized by a left ventricular or biventricular dilation and systolic dysfunction in the absence of a coronary artery disease or overload [99]. Ankrd1 and CTGF are well-known YAP/TAZ transcriptional targets. The mRNA and protein levels of the YAP/TAZ are significantly increased in human hearts with idiopathic dilated cardiomyopathy (IDC), as well as desmin-associated cardiomyopathy (DES) in mice hearts, and accumulate in the CMs nucleus in patient and mouse hearts, while the expression levels of ANKRD1, CTGF, and CYR61 are significantly upregulated [100]. In addition, studies have shown that Ankrd1 is associated with HCM and IDC [101], and CTGF regulates the expression of ECM-related genes and promotes cardiac remodeling and fibrosis in a mouse model of DCM [102]. Furthermore, the transdifferentiation of cardiac fibroblasts to myofibroblasts is a key event in cardiac matrix remodeling [103,104,105]. However, some studies have shown that the YAP/TAZ signaling pathway can block the transdifferentiation of myofibroblasts in the DCM matrix remodeling. A YAP/TAZ deficiency results in reduced matrix remodeling and shrinkage, but the mechanism is unclear [106]. These data suggest that both the YAP/TAZ may ultimately target interstitial fibrosis in the pathological development of DCM, but whether the YAP/TAZ contributes to CMs degeneration remains unclear.

AC is a genetic disorder characterized by cardiovascular events following ventricular tachycardia or fibrillation [107]. Studies have shown that YAP/TAZ is involved in the development of AC pathology and adipogenesis. Neurofibromin type 2 (NF2) is an upstream molecule of the Hippo pathway that localizes to intercalated discs (IDs). NF2 can interact with β-catenin, protein kinase C-α (PKC-α), and cadherin. In AC, NF2 is activated, resulting in Hippo kinase cascade phosphorylation, the inactivation of the YAP, and enhanced adipogenesis [108]. Another important regulatory mechanism regulates the YAP activity independently of Hippo kinase. This process involves the binding of the YAP to α-catenin and its subsequent dissociation at the cell membrane [109,110]. Furthermore, the activation of the Hippo pathway in adipogenesis may be related to its inhibitory effect on canonical WNT signaling. The YAP can bind to β-catenin and the secreted glycoprotein Dickkopf-1 (DKK-1), thereby sequestering the YAP and β-catenin, inhibiting the canonical Hippo and Wnt pathways and promoting the cell differentiation towards adipogenesis [111]. Increasing the YAP activity will enhance osteogenic differentiation and inhibit lipogenic differentiation, while the transdifferentiation and osteogenic differentiation of VSMCs have a significant contribution to vascular calcification [112,113]. These pathological changes inhibit the WNT pathway by activating the YAP pathway, increasing the fat formation, providing a new pathophysiological mechanism for arrhythmogenic cardiomyopathy, and may become a new target for the treatment of this disease.

In addition to primary cardiomyopathy, cardiomyopathy in a broad sense also includes specific cardiomyopathy with a clear etiology; it is also associated with systemic diseases. Diabetic cardiomyopathy, which is markedly characterized by cardiac hypertrophy and an impaired systolic and diastolic function [114,115], has been identified as a microvascular complication, and cardiac microvascular endothelial dysfunction is a major contributor to diabetic cardiomyopathy. Diabetic cardiomyopathy is the first link in the development of lesions and is present during the entire process [116,117]. Studies have shown that the renin-angiotensin system (RAS) and mitogen-activated protein kinase (MAPK) pathways regulate the YAP activity, while the (pro)renin receptor (PRR) is a key RAS membrane receptor. Under high glucose conditions, the expression levels of the PRR and YAP are increased in the myocardium, and changes in the PRR expression can induce the same changes in the YAP in DCM rats and high glucose rats. In addition, the YAPs blockade attenuates the accelerated development of myocardial fibrosis [118,119]. Another feature of diabetic cardiomyopathy is cardiac interstitial and perivascular fibrosis, which accelerates the diastolic dysfunction, a pathological mechanism that is closely related to the ECMs deposition [120]. It was found that lncRNA MALAT1 is widely expressed in CMs under high glucose, and lncRNA MALAT1 positively regulate the nuclear translocation of the YAP by binding to the cAMP response element-binding protein (CREB), thereby increasing the inflammatory response and collagen accumulation in cardiac fibroblasts and diabetic cardiomyopathy mice in a high-glucose microenvironment [121].

**Figure 3:**
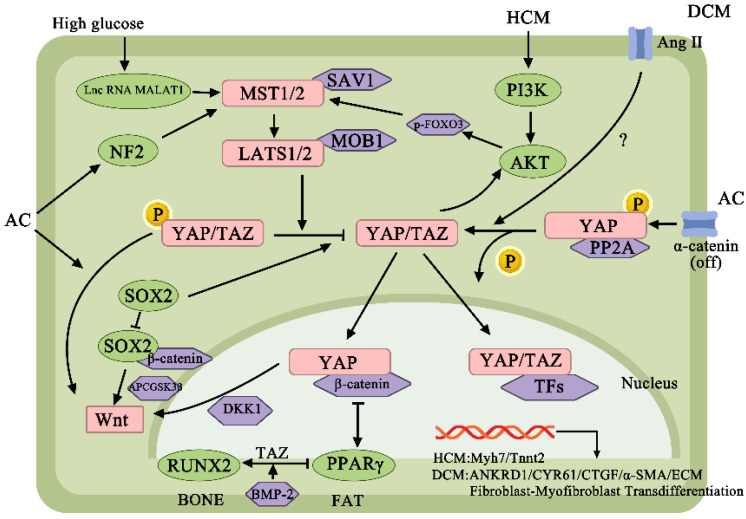
YAP/TAZ signaling proteins in cardiomyopathy. SAV1, salvador homolog-1; MOB1, MOB kinase activator 1; p-FOXO3, phospho-forkhead box O3; SOX2, SRY-box transcription factor 2; ADCGSK3β, glycogen synthase kinase-3β; BMP-2, bone morphogenetic protein 2; PP2A, protein phosphatase 2A; α-SMA, α-smooth muscle actin.

In cardiomyopathy, the YAP/TAZ is often overexpressed and leads to a poor prognosis, but the mechanism is complicated, and research on the YAP/TAZ in cardiomyopathy still needs more exploration. Figure 3 shows the YAP/TAZ signaling proteins in cardiomyopathy.

## 5. YAP/TAZ in Aortic Disease

The largest artery in the human body is the aorta, and related diseases mainly manifest as a tumor-like dilatation, dissection, and developmental malformation. It is a complex disease caused by various factors, such as the environment, behavior, and genes [122]. Aortic dissection (AD) is one of the most elusive vascular diseases with a high mortality, and a Stanford type A aortic dissection (STAAD) is involved in the ascending aorta [123,124]. Studies have shown that ECM disturbances and the increased apoptosis in VSMCs are associated with the downregulation of the YAP in human STAAD samples, while the downregulation of the YAP and the increase in VSMCs apoptosis over time during the ECM injury were verified in a STAAD mouse model, which indicates that the YAPs downregulation due to a mechanical stress disruption was associated with the development of STAAD by inducing apoptosis in aortic VSMCs [125]. In fact, many YAP/TAZ-related sites are involved in regulating the VSMCs function. For example, the inhibition of TEAD can reduce the proliferation of VSMCs or interact with the cardiac protein-serum response factor (SRF) complex to promote the transformation of VSMCs from a proliferative phenotype to a contractile phenotype [126,127,128]. Similarly, inflammatory infiltrated and activated valve interstitial cells (VICs) can also synthesize the fibrotic extracellular matrix, resulting in the valve thickening and stiffness and calcified mineral deposition [129]. The aldehyde-ketoreductase family 1 member B (Akr1B1) has been recognized as one of the potential therapeutic targets for the calcification of VICs. Studies have shown that the protein level of YAP is positively correlated with Akr1B1. The YAP can activate the RUNX2 promoter by binding to TEAD1, and inhibiting the YAP reverses the upregulation of RUNX2 induced by the overexpression of Akr1B1 [130].

Inflammatory cells infiltrate blood vessels, while VSMCs change phenotype and secrete a large number of metalloproteinases to degrade the ECM. The phenotype of VSMCs plays an important role in the balance of the ECMs synthesis and degradation. In conclusion, YAP/TAZ protein signaling is important for maintaining the contractile phenotype of VSMCs.

## 6. Future Prospects for Clinical Applications

YAP/TAZ is involved in the regulation of many key physiological processes, such as cell proliferation and differentiation, embryonic development, and tissue regeneration. The treatment methods for the YAP/TAZ have been recommended for the treatment of various diseases. The mechanism of the YAP/TAZ is not only a single pathway, but the multiple targeted intervention of the YAP/TAZ is very attractive. Targeting the YAP/TAZ-TEAD complex, kinases, and the upstream and downstream signaling that regulates the YAP/TAZ are all ideal approaches [43,131,132].

Various upstream signals can regulate the YAP/TAZ localization by activating the MST-LATS kinase cascade. XMU-MP-1 is a reversible and selective MST1/2 inhibitor that activates the downstream effectors YAP/TAZ to promote cell growth. XMU-MP-1 attenuates a myocardial mitochondrial dysfunction and CMs necrosis in a mouse model of myocardial ischemia/reperfusion (I/R) injury and inhibits inflammatory responses by stimulating the I/R M2 macrophage polarization [133]. In a mouse model of diabetic cardiomyopathy, glycemic control and the use of XMU-MP-1 reduce the CMs apoptosis and improve the myocardial function in diabetic mice [134]. TIR/BB-loop mimetic AS-1 can reduce myocardial hypertrophy under a pressure overload by increasing the phosphorylation [135]. While the loss of the WWC protein expression in mouse liver can lead to a tissue overgrowth, inflammation, fibrosis, and the formation of liver cancer by reducing the LATS activity, the expression of the WWC protein may be considered as a new prognostic factor for human hepatocellular carcinoma. As a WWC-derived protein was designed, SuperHippo can inhibit the YAP/TAZs activity by inducing the phosphorylation of LATS1/2 [136].

In addition, the YAP/TAZs activity can also be interfered from a pharmacological inhibition and genetic inhibition. A new type of YAP chemical inhibitor CA3 has been proved to have strong anti-tumor activity against esophageal adenocarcinoma [137]. In the mouse hepatocellular carcinoma model, treatment with YAP which targeted small interfering RNA lipid nanoparticles (siRNA LNP) can lead to a significant tumor regression [138]. In non-neoplastic diseases, some studies also showed that an intravitreal injection of the YAP siRNA significantly inhibited choroidal neovascularization and neovascularization in oxygen-induced retinopathy mouse models [139]. In addition, a study has shown that inhibiting the expression of the YAP in mouse cardiomyocytes by adeno-associated virus 9 (AAV9) can significantly improve the cardiac function after a myocardial infarction [140]. AAV9 can be regarded as safe for treatment in humans due to its minimal immune response; this may suggest that this method can control the YAP pathway for a clinical application. An antisense oligonucleotide (ASO) called ION537 targeting YAP1 is now entering clinical trials, which is an important milestone in targeting YAP therapy [141].

However, due to the specific structural properties of the YAP/TAZ, targeting the the YAP/TAZ-TEAD complex may be the better strategy. Verteporfin, for example, is a classic YAP inhibitor and autophagy inhibitor that disrupts the YAP-TEAD interaction and induces apoptosis [142,143,144]. Studies have shown that the targeted inhibition of the YAP/TAZ through the lung-specific and controlled release of verteporfin ameliorates pulmonary hypertension in rats [145]. Gap19, a Cx43 mimetic peptide, has also been shown to target YAP signaling in reactive astrocytes after an intracerebral hemorrhage injury to reduce the expression of proinflammatory cytokines (IL-1β, IL-6, TNF-α, and MCP1), increase anti-inflammatory cytokines (IL-4 and IL-10), and increase the neuronal cell survival. Verteporfin reversed the anti-inflammatory effects of Gap19 in vitro [146,147]. Verteporfin attenuated vascular damage in a model of Ang II-related hypertensive kidney injury and reversed the expression of the TNF-α, IL-1β, and monocyte chemotactic protein 1 (MCP1) caused by increased Ang II levels [148]. In addition, verteporfin is commonly used as a selective vaso-occlusion therapy for choroidal vascular abnormalities by sensing the expression of ox-LDL receptors that preferentially accumulate in abnormal neovascular ECs [149,150,151]. Flufenamic acid is also a common YAP/TAZ-TEAD complex inhibitor that can bind to TEAD2 and inhibit the TEADs function and TEAD-YAP-dependent processes. There have been case reports of an acute inferior MI in patients with Kounis syndrome following the intramuscular injection of 1g of etofenamate [152]. In a mouse model of cardiac hypoxia/reperfusion, flufenamic acid reduced arrhythmias, such as early afterdepolarization by targeting the transient receptor potential vanilloid 4, an intracellular Ca^2+^-activated channel [153]. In recent years, studies have shown that Vestigial-like family member 4 (VGLL4) acts as a transcriptional repressor to inhibit a YAP-induced cell proliferation in humans, and it plays a transcriptional regulatory role by pairing its own Tondu domain with TEADs [154,155]. TEAD1 inhibits the CMs proliferation by binding the VGLL4 protein to inhibit the TEAD1-YAP interaction [156]. VGLL4 can also target the Hippo-YAP/TEAD1 signaling pathway by inducing the degradation of TEAD1 to attenuate oxidative stress, inflammation, and dysfunction in HUVECs induced by ox-LDL [157]. In addition, VGLL4 promotes pulmonary hypertension and pulmonary arterial remodeling through a signal transducer and the activation of transcription 3 (STAT3) signaling [158]; alternatively, the specific knockout of VGLL4 in ECs increases the YAP expression and heart valve malformation [159].

The latest research found that palmitoylation is an important protein modification mode of TEAD binding with the YAP/TAZ, and its unique binding mode makes the TEAD easy to target [160]. VT3989 and IK-930, two TEAD palmitoylation inhibitors, have been studied in human clinical trials [161,162,163,164]. Table 2 shows potential drugs targeting YAP/TAZ and its upstream and downstream pathways in pre-clinical trials and clinical trials.

## 7. Discussion and Conclusions

In recent years, scientific research on the complex molecular mechanisms of the YAP/TAZ and its related signaling pathways has grown exponentially, but the current research on the YAP/TAZ in CVDs is still in the exploratory stage [165]. We believe that the upstream and downstream targeting of YAP/TAZ signaling proteins and mediating cross-linking between different pathways should be focused on. Inflammatory stimuli, ECM remodeling, mechanical traction, cell adhesion, cell polarity, the signals activated by G protein-coupled receptors, and an altered cellular metabolism can be involved in regulating the YAP/TAZs activity [166,167]. The stimulation of inflammation and ECM remodeling should be more worthy of attention. In particular, the mechanism of YAP/TAZ in cardiac repair, macrophage polarization, and immune cell-mediated cardiac inflammatory responses after an MI is not yet clear. Overall, a complex and vast network of intrinsic and extrinsic signals is intertwined in a connected network, but the mechanisms are still largely unknown and are worth exploring in the future.

Second, the activation of the YAP/TAZ can be protective or damaging in different CVD models. Even in the same disease model, protective/injurious effects occur due to different expression levels. For example, studies have shown that the YAP can have a protective effect by promoting the CMs proliferation [168], while studies have also shown that the overexpression of wild-type YAP exerts deleterious effects by promoting CMs hypertrophy [169]. Due to the different experimental designs and the different methods, these research results are worthy of analysis and reference. The most notable concern is that the phosphorylation status of the YAP may determine its impact on different CVDs. Dissecting the different roles of the YAP/TAZ in different forms and degrees of expression may provide better targeted strategies.

Furthermore, different cell types may respond to the YAP/TAZ to varying degrees. Thus, what may be considered low YAP/TAZ activity in one particular cell type may be high activity in another cell type. The interactions between the different cells targeted by the YAP/TAZ should be further examined.

It is reasonable to target the YAP/TAZ in heart disease, but there are still important considerations before it undergoes the transition to clinical application. These questions include the specificity of the cell type of conduction, the possibility of being out of target in non-heart organs or diseases under the condition that the physiological effects of YAP disease have been clarified, off-target effects, and the best way and duration of a YAP/TAZ intervention.

It is worth noting that tumorigenic stimulation is a difficult problem in researching YAP/TAZ signaling. Elevated YAP/TAZ activity and YAP/TAZ nuclear enrichment have been reported in many types of human cancers, such as colon, lung, breast, liver, and gastric cancer [170,171,172,173,174]. The biological and functional mechanisms of YAP/TAZ signal proteins in patients with different diseases are different. Studies have shown that cells that are no longer in their original microenvironment but maintain high YAP levels (e.g., after the transition from a stiff ECM to a more flexible ECM or after inflammation/tissue repair subsides) may have a protective effect, and YAP/TAZ may switch from a tumor inducer to a tumor suppressor [175]. However, heart cancer is very rare. The unique tissue and cell composition of the heart limits the possibility of cancer cell accumulation. Due to the highly conservative nature of the Hippo signaling pathway, the YAP/TAZ rarely causes human cancer through a somatic mutation. The function of the YAP is related to the type of disease and the expression form of the YAP itself. In non-cancer diseases, studies have shown that increasing the YAP activity in various organs can reprogram fully differentiated cells into an undifferentiated state in vivo [176,177,178]. Even in cancers, Pearson and other researchers have confirmed that pan-cancer can be classified according to the different expressions of the YAP and the YAP responsive adhesion regulator [179]. The YAP/TAZ have a wide range of functions, but also lacks specificity. Targeting the YAP can improve the pathological status and may also lead to a physiological disorder.

The YAP/TAZ protein participate in the physiological process of cardiovascular disease by maintaining cell proliferation, transmitting mechanical stimulus signals, promoting angiogenesis, and regulating the immune response [180,181]. Studies have shown that the YAP/TAZ can alleviate a myocardial infarction injury by maintaining cardiomyocyte proliferation and survival, while regulating inflammation, fibrosis, and angiogenesis after a heart injury [182,183,184]. Targeting the form specificity and tissue specificity of the YAP in specific diseases is a promising therapeutic strategy. We should weigh the pros and cons when clarifying the pathways and targets of YAP/TAZ signaling in more experimental studies and should examine the positive effects of the YAP/TAZ in the treatment of CVDs while minimizing the risk of its carcinogenic properties and other disadvantages.

In conclusion, the potential targeting of YAP/TAZ signaling for clinical indications of CVDs in the future has been fully described, and our understanding of the YAP/TAZ still needs a further exploration.

## Figures and Tables

**Table 1:** Targeting different cells or cellular constituents of YAP/TAZ signaling proteins in myocardial infarction. ↑, Increased; ↓, decreased.

	Participant	Pathway	Expression of Nuclear YAP	Biological Function	References
CM	miR-199a-3p	Direct target Hippo pathway proteins TAOK1 and E3 ubiquitin ligases β-TrCP.	↑	Activate YAP, regulate actin cytoskeleton, and promote cardiomyocyte proliferation.	[35]
	miR302-367 cluster	Targeting multiple components of the Hippo signaling kinase cascade.	↑	Promote cardiomyocyte proliferation.	[36]
	LncDACH1	Direct binding to PP1A induces YAP1 phosphorylation.	↓	Inhibits cardiomyocyte mitosis and cardiac regeneration.	[37]
	miR-206	PI3K-independent mechanism.	↑	Increases cardiac hypertrophy and survival.	[39]
	miR-29	Targeting miR-29 3′ UTR to inhibit PTEN, thereby inactivating mTOR pathway to inhibit cell growth.	↑	Down regulation of miR-29 contributes to cardiac fibrosis.	[43,44]
	miR-484	Direct target miR-484 to YAP1 3′UTR and inhibit mitochondrial fission.	↓	Knockdown of Yap1 inhibits the viability of H9c2 cells and induces apoptosis and inflammation.	[45]
	miR-411	Regulate Foxo1 expression and induce YAP activity.	↑	It protects myocardial cells from excessive apoptosis caused by hypoxia and oxidative stress during MI, thereby limiting infarct size and cardiac remodeling.	[80]
	miR-9	(-)	↓	Knockdown of Yap1 inhibits cell proliferation and promotes apoptosis of H9c2 cells.	[81]
EC	unidirectional shear flow	activates integrin and promotes integrin-Gα13 interaction, leading to RhoA inhibition and YAP phosphorylation and suppression.	↓	Inhibition of YAP/TAZ can reduce the attachment and infiltration of monocytes and alleviate the progress of atherosclerosis.	[20]
	unidirectional shear flow	Reduce JNK signal conduction through YAP/miR-206/PDCD4 signal pathway.	↑	It inhibits the apoptosis of myocardial microvascular endothelial cells, reduces the adhesion of monocytes on endothelial cells, and inhibits the formation of atherosclerosis.	[20,49]
	HSPA12B	SPA12B—YAP synergy	↑	Regulation of endothelial cell proliferation, migration and angiogenesis after hypoxia.	[47]
	miR-93	Inactivate the Hippo/Yap pathway.	↑	Reduced HUVEC apoptosis and increased HUVEC migration and angiogenic capacity.	[50]
Hormone	Progesterone	Progesterone receptor-dependent manner.	↑	Promotes proliferation of neonatal and adult cardiomyocytes.	[57]
	Melatonin	Activation of the melatonin receptor inhibits miR-143-3p.	↑	promotes cardiomyocyte proliferation and cardiac regeneration.	[58]
Fibroblast	Erbb2	Cytoskeleton remodeling and phosphorylation of ERBB2-ERK-YAP mechanotransduction.	(-)	Changes that promote epithelial-mesenchymal transition (EMT-like), including production of substantial ECM and cytoskeletal remodeling, and CM migration.	[61]
	DGC partially disassembled	DGC binds to DGC via Dag1 and reduces its stability, decomposes myofibrils and activates YAP and ERK.	↑	Promotes division of induced pluripotent stem cells from mice and humans into cardiomyocytes.	[62]
	Spatial changes in collagen organization	Mechanically activated p38-YAP-TEAD signaling (independent of the Lats1/2-Hippo pathway).	↑	Modulation of cardiac fibroblast phenotype.	[65]
	Rigid matrix	ECM-AT1R-YAP.	↑	High expression and nuclear localization of YAP in cardiac fibroblasts promotes CF activation.	[66]
	Fibroblast-specific deletion of YAP/TAZ	Regulate the proliferation and transdifferentiation of fibroblasts into muscle fibroblasts, and act on Wnt and TGF- β Signal pathway.	↓	Reduced fibrotic and fibroinflammatory responses and improved cardiac function after MI.	[67]
ImmuneCell	M1-macrophages	Binds to TBS or modulates IL6 through the Tak1-p38 MAPKs signaling pathway, interacts with the HDAC3-NCoR1repressor complex.	↓	Reduces pro-inflammatory responses, cardiac fibrosis and protects cardiac function after MI, suppresses repair macrophage phenotype.	[71]
	Tregs	Recruit Tregs.	↑	Rescue Tregs infiltration into YAP/TAZ mutant injured myocardium and reduce fibrosis.	[74]
	intersects with CM	Attenuates TLR4/NF-κB signaling.	↑	Knockout of YAP in the heart exacerbates lipopolysaccharide-induced cardiac shock.	[75]
	IRF3	YAP blocks the dimerization and nuclear translocation of transcription factor IRF3.	↓	YAP deficiency leads to innate immune enhancement.	[77]
	TAK1	YAP directly binds with TAK1 and IKKs complex to inhibit- κ Inflammatory response triggered by B signal.	↑	Regulate the expression of matrix degrading enzymes and weaken chondrolysis.	[78]
	TBK1	YAP directly inhibits TBK1 activity through the transactivation domain.	↓	YAP/TAZ deletion alleviates TBK1 inhibition and enhances antiviral response.	[79]

**Table 2:** Potential drugs targeting YAP/TAZ and its upstream and downstream pathways in pre-clinical trials and clinical trials.

Target	Participant	State	Disease	Function	References
Core kinase	XMU-MP-1	Preclinical	Myocardial hypoxia reperfusion	Mitigating myocardial I/R injury and preserving myocardial function in mice.	[133]
			Diabetes cardiomyopathy	Combining insulin therapy improves myocardial function and reduces apoptosis in diabetes mice.	[134]
			Cerebral ischemia-induced brain injury	Inhibiting the expression of phosphorylated STAT3 in rats.	[147]
	AS-1	Preclinical	Cardiac hypertrophy	Inhibiting paracrine secretion of cardiac fibroblasts to improve cardiac hypertrophy in mice.	[135]
	WWC-derived protein	Preclinical	Hepatocellular carcinoma	Reducing LATS activity and leading to liver cancer in mice.	[136]
YAP/TAZ	CA3	Preclinical	Esophageal adenocarcinoma	Reducing tumor sphere formation of human esophageal adenocarcinoma cells and making drug-resistant esophageal adenocarcinoma cells sensitive to radiotherapy.	[137]
	siRNA/shRNA	Preclinical	Hepatocellular carcinoma	Targeting YAP restores hepatocyte differentiation and causing pronounced tumor regression in a genetically engineered mouse HCC model.	[138]
			Ocular neovascular diseases	Knockdown of YAP inhibits pathological ocular neovascularization in mice.	[139]
			Myocardial infarction	Reducing myocardial injury and improving cardiac function in mice.	[140]
	ASO (ION537)	Clinical	Solid tumor	Preventing the production of YAP1 protein by binding to the complementary region of YAP1 mRNA.	[141]
YAP-TEADs	Verteporfin	Clinical	Pulmonary hypertension	Improving pulmonary vascular remodeling by delivering drug particles in rats.	[145]
			Intracerebral hemorrhage	Reversing the anti-inflammatory effect in vitro and blocking the protective effects of Gap19 in vivo after intracerebral hemorrhage in mice.	[146]
			Cerebral ischemia-induced brain injury	Aggravating oxygen-glucose deprivation/reperfusion damage induced by STAT3 signaling in rats.	[147]
			Hypertensive nephropathy	Alleviating proteinuria and kidney damage caused by angiotensin II in mice.	[148]
			Angiogenesis-related ophthalmopathy	Interacting with oxygen and/or biological substrates and producing vascular endothelial cell damage that leads to platelet aggregation, activation of the clotting cascade and microvascular occlusion.	[149,150,151]
	Flufenamic acid	Preclinical	Arrhythmia	Reducing frequency of afterdepolarizations in mice.	[153]
	VGLL4	Preclinical	Pulmonary hypertension	Upregulating acetylation and promoting pulmonary artery remodeling through STAT3 signal transduction in mice.	[158]
			Valvular heart disease	Balancing the proliferation and apoptosis of valve interstitial cells by competing with YAP-TEAD complex and maintaining the normal shape and function of the valve in mice.	[159]
	IK-930	Clinical	Solid tumor; mesothelioma	Selectively inhibiting TEAD-dependent transcription by directly blocking autopalmitoylation.	[163]
	VT3989	Clinical	Solid tumor; mesothelioma	Forming a covalent bond rapidly and selectively with a conserved cysteine located within the unique deep hydrophobic palmitate-binding pocket of TEADs.	[161,164]

## Data Availability

Not applicable.
